# Patient-Centered, Sustainable Hypertension Care: The Case for Adopting a Differentiated Service Delivery Model for Hypertension Services in Low- and Middle-Income Countries

**DOI:** 10.5334/gh.978

**Published:** 2021-09-02

**Authors:** Rebecca L. Tisdale, Danielle Cazabon, Andrew E. Moran, Miriam Rabkin, Helen Bygrave, Jennifer Cohn

**Affiliations:** 1 Stanford Health Policy, Centers for Health Policy/Primary Care and Outcomes Research, Department of Medicine, Stanford University School of Medicine, Stanford, California, US; 2 Veterans Affairs Palo Alto Health Care System, Palo Alto, California, US; 3 Resolve to Save Lives, an initiative of Vital Strategies, New York, US; 4 Department of Medicine, Columbia University, New York, US; 5 ICAP at Columbia University, Columbia University Mailman School of Public Health, New York, US; 6 Department of Epidemiology, Columbia University Mailman School of Public Health, New York, US; 7 International AIDS Society, Geneva, CH; 8 Division of Infectious Diseases, University of Pennsylvania School of Medicine, Philadelphia, Pennsylvania, US

**Keywords:** Hypertension, health systems, health policy, HIV/AIDS, chronic disease

## Abstract

Expanding hypertension services in low- and middle-income countries requires efficient and effective service delivery approaches that meet the needs and expectations of people living with hypertension within the resource constraints of existing national health systems. Ideally, a hypertension program will extend treatment coverage while maintaining service quality, maximizing efficient resource utilization and improving clinical outcomes. In this article, we discuss lessons learned from HIV differentiated service delivery initiatives, and make the case that the same approach should be adopted for hypertension programs.

## Background

Hypertension is the leading single cause of cardiovascular disease (CVD) worldwide, responsible for more deaths than all infectious diseases combined in the pre-COVID era [1,2]. Globally, hypertension prevalence is growing most rapidly in low- and middle-income countries (LMICs) [3], reflecting aging populations and a rising risk factor prevalence. Approximately 17.5% of adults in LMICs are living with hypertension, of whom only 39% have had their hypertension diagnosed, just 29% are being treated, and a mere 10% have well-controlled blood pressure (BP) [4].

Controlling hypertension is a public health priority, but health systems in many LMIC are not on track to reach the World Health Organization (WHO) goal of 25% reduction in raised BP by 2025 [5]. In order to achieve global and national targets, a new approach is required that delivers efficient and effective hypertension services at scale in resource-constrained settings.

We propose that key lessons can be learned from a service delivery approach used for HIV, another chronic condition [6]. Both hypertension and HIV require primary prevention services, screening and diagnosis, linkage to care, lifelong behavioral and biomedical interventions, ongoing clinical and laboratory monitoring, and support for long-term medication adherence [7]. Yet the scale-up of HIV services has been considerably more effective, with 81% of people living with HIV (PLHIV) aware of their diagnosis, 67% on treatment, and 59% achieving viral suppression [8]. In addition to robust funding and political commitment, HIV programs have benefited from innovative service delivery models that can be adapted for other chronic conditions [9].

At the heart of successful HIV scale-up is a *public health* approach, including the use of simplified treatment algorithms that enable standardized national training programs, task sharing among health care workers, decentralized clinical and laboratory services largely delivered at the primary care level, streamlined procurement of drugs and commodities, and consistent monitoring and evaluation processes [10]. More recently, the public health approach to HIV programming has adopted the principles of *differentiated service delivery* (DSD), a strategy in which service delivery models are tailored for different groups of PLHIV according to their clinical characteristics, specific population type and context. The goal of DSD is to increase service coverage, quality and efficiency, provide person-centred care, and improve outcomes, within the context of resource and health system limitations [11].

While initially developed in the context of HIV, DSD was conceptualized from the start to apply to any chronic disease, including hypertension [12,13,14]. In this article, we make the case for applying the DSD model to hypertension services and outline the steps required to implement this strategy.

## DSD: Lessons from HIV and the case for extending them to hypertension

**Figure 1:**
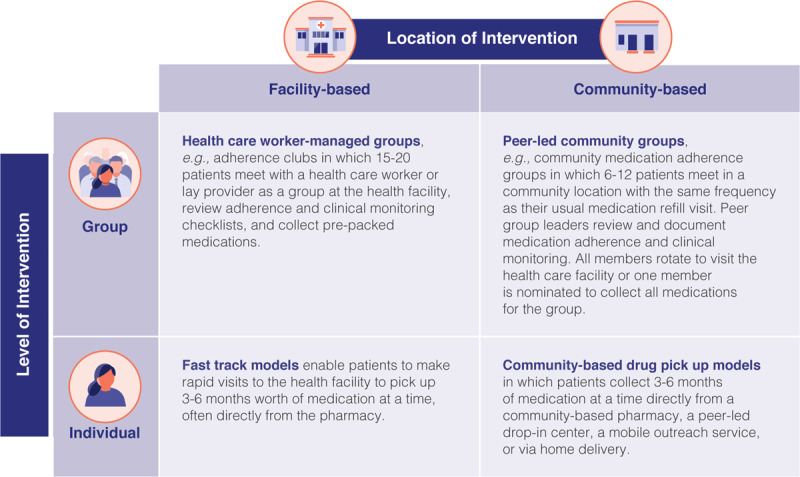
Illustrative DSD models for people doing well on treatment.

DSD is a patient-centered approach that simplifies and adapts health services to reflect the preferences and expectations of recipients of care, while reducing unnecessary burdens on patients and the health system [15]. DSD focuses less on the ‘what’ of service delivery and more on the ‘how’ – specifically on how to optimize the delivery of clinical, laboratory, pharmacy and psychosocial support services using four building blocks: [15] *where* (service location), *when* (service frequency and time), *who* (health worker cadres), and *what* (the package of services). For example, services for people established on HIV treatment are designed to maximize both choice and efficiency, with less frequent clinical and pharmacy visits and services delivered either at health facilities or in the community by a wide range of health workers, including peers and/or lay health workers. In contrast, services for people with more complex or advanced disease may be delivered primarily at health facilities, with more frequent visits, by more highly trained healthcare workers. Figure 1 depicts illustrative DSD models widely used for individuals established on HIV treatment.

A growing body of evidence supports the effectiveness of DSD for improving HIV cascade outcomes while controlling or reducing cost [11,16,17]. DSD also promoted health system and patient resilience during the COVID-19 pandemic: [18,19] efficiency, decentralization of services, and streamlined interactions with the health system were ever more critical when travel was curtailed, clinics were closed, and financial and human resources were shifted away from chronic disease services [20].

The hypertension cascade data make it clear that the *status quo* service delivery models are not serving the needs of people living with hypertension. The capacity of the primary health care system to manage chronic care of any kind is limited in most LMICs, limiting access to diagnostic and treatment services and defeating efforts to control many non-communicable diseases. Considering the even larger cohort of patients in need of hypertension care, pivoting to a public health approach, and more specifically to a DSD strategy, may enable countries to leverage lessons from HIV programs to improve hypertension diagnosis, linkage to treatment, and control of blood pressure at scale.

## DSD for hypertension: What would it take?

In theory, DSD models developed for ART delivery and described in Figure 1 should translate well to hypertension and may address several of the critical barriers to the scale-up of hypertension services in LMICs [21,22]. Less-intensive models that de-link medication dispensing from clinical examination for people established on treatment are more convenient for people with hypertension and more efficient for overburdened health facility staff. Enabling task-shifting to non-physician clinicians and laypeople can expand the geographic coverage and reach of hypertension services.

There is an emerging body of evidence for the use of differentiated hypertension models. For example, DSD models have been designed for people with both HIV and hypertension in Eswatini, using a facility-based group model [23]. A pilot project in Kenya also used a facility-based group model for PLHIV and for people with hypertension and diabetes and found the model feasible, efficacious, and acceptable to both patients and healthcare workers [24,25]. A pilot of hypertension adherence and treatment clubs in Nigeria is underway [26], and a multicomponent intervention focused on home visits by community health workers (CHWs) has already demonstrated significant improvement in hypertension control in rural communities across several South Asian countries in a randomized controlled trial [27]. But what will it take to deliver DSD-for-hypertension at scale?

**Figure 2:**
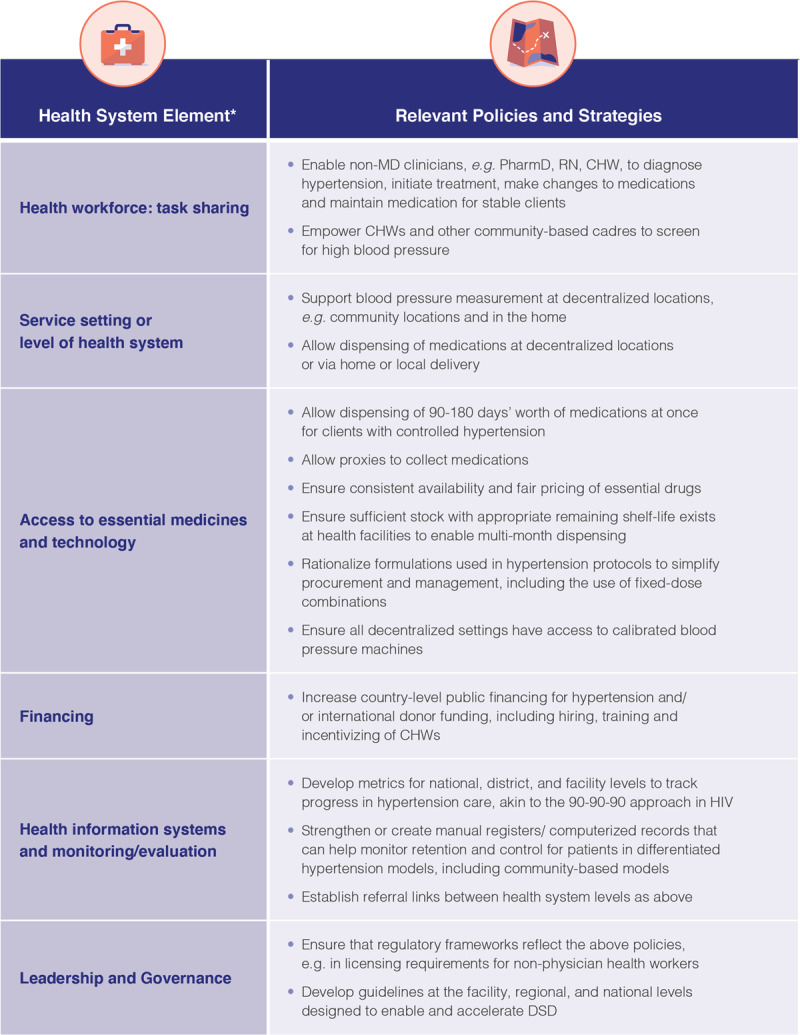
Key Steps Necessary for DSD in Hypertension. * Organized into WHO health systems building blocks [35].

Moving a new model to scale requires supportive government policies and guidelines along with champions on both the ‘supply side’ (the health system) and the ‘demand side’ (recipients of care and communities). And while DSD models can be more efficient than standard models, achieving broad scale coverage will nonetheless require increased funding for hypertension services. Figure 2 lays out key steps necessary to adopt DSD for hypertension.

An assessment of current policies can help countries understand the present policy environment, and the changes necessary to enable DSD approaches to care. Existing global hypertension programs, such as those of WHO-HEARTS and Resolve to Save Lives, an initiative of Vital Strategies, already involve many of these components and may be able to provide technical support for incorporating DSD and its health systems framework [28,29,30].

Countries and health systems preparing to commit to DSD must also make the policy and business case for this approach. In some cases, more evidence is needed to do so. How are we to gather this evidence?

First, standardized targets, indicators and monitoring and evaluation (M&E) frameworks are essential. The UNAIDS 95-95-95 goals for HIV urge each country to ensure that 95% of people living with HIV know their HIV status [31], 95% of those individuals receive ART, and 95% of those on ART achieve sustained viral suppression. Similar bold and defined targets can be developed for the hypertension care cascade, which can help drive and refine innovative models of care, such as DSD. Clear indicators to define the number of people diagnosed with HTN, linked to treatment, retained in care, and achieving BP control must be part of the implementation of DSD models for HTN. When paired with quality improvement responses, ongoing impact assessments can accompany implementation and scale-up and identify the challenges to meeting these targets that will inevitably arise [32].

The design of additional studies will depend on evidence gaps identified. These can include mixed methods implementation studies to document quality, coverage, impact, and acceptability, qualitative explorations of user and health care worker experiences, time-motion studies to estimate health workforce need and to identify areas for increasing efficiencies, and costing and economic analyses, including mathematical models to assess cost-effectiveness or help in budget planning [33,34].

## Conclusion

The epidemic of hypertension in LMICs demands immediate action to slow and reverse the growing burden of cardiovascular disease. However, interventions must take a public health approach, and be tailored both to health system capacity and to the needs of people with hypertension. Adopting a DSD approach for hypertension has the potential to expand treatment coverage, improve BP control, maintain or improve quality of care, and make the most of limited health care resources.
